# Reduced emotional empathy in adults with subclinical ADHD: evidence from the empathy and systemizing quotient

**DOI:** 10.1007/s12402-017-0236-7

**Published:** 2017-08-23

**Authors:** Y. Groen, A. E. den Heijer, A. B. M. Fuermaier, M. Althaus, O. Tucha

**Affiliations:** 1 Faculty of Behavioral and Social Sciences, Clinical and Developmental Neuropsychology, University of Groningen, Grote Kruisstraat 2/1, 9712 TS Groningen, The Netherlands; 2 Child and Adolescent Psychiatry, University of Groningen, University Medical Center Groningen, Groningen, The Netherlands

**Keywords:** ADHD, Adults, Social cognition, Emotional, Empathy

## Abstract

Studies in children with ADHD suggest impairments in social cognitive functions, whereas studies in adults with ADHD are scarce and inconclusive. The aim of this study was to investigate the relationship between ADHD traits and self-reported social cognitive style in a sample of adults from the general population. For this purpose, a community sample of 685 adults filled out online self-report questionnaires about ADHD symptoms (ADHD Rating Scale, ARS), social cognitive functioning and friendships. The Empathy Quotient (EQ) with the subscales Cognitive Empathy (CE), Emotional Empathy (EE) and Social Skills (SS), and the Systemizing Quotient (SQ) were included for measuring social cognitive style and the Friendship Questionnaire (FQ) for the quality of friendships. Participants who met the DSM-5 criteria on the ARS (‘subclinical ADHD’; *n* = 56) were compared regarding their social cognitive functioning scores with a control group (*n* = 56) that was matched for age, sex and student status. With small effect sizes, the subclinical ADHD group showed reduced EE scores on the EQ and a more male social cognitive profile. This result was not influenced by sex or ADHD subtype. This study points to a relationship between traits of ADHD and the emotional aspect of empathy, whereas more complex aspects of empathy were unrelated. These findings should be corroborated in clinical patients with ADHD, employing neuropsychological tests rather than self-report questionnaires.

## Introduction

Attention deficit hyperactivity disorder (ADHD) is not only associated with symptoms of inattention, impulsivity, impaired inhibition and hyperactivity (American Psychiatric Association, APA 2013) but also associated with a range of (psycho-)social problems across the lifespan (Asherson et al. 2007; Kooij et al. 2010; Canu and Carlson 2007). Children with ADHD often demonstrate problematic peer functioning, including peer rejection and social isolation (De Boo and Prins 2007; Hinshaw 2002; Hoza et al. 2005), that may be a result of inappropriate social behavior such as frequently interrupting and not listening to others carefully (Guevremont and Dumas 1994). Several studies have, however, related the difficulties in social and interpersonal functioning in ADHD to traits of autism spectrum disorders (ASD) (Nijmeijer et al. 2008), a disorder that is characterized by specific impairments in empathy (Baron-Cohen and Wheelwright 2004; Wheelwright et al. 2006). Part of the childhood population of patients with ADHD have been shown to present problems in social cognition similar to those in ASD and have been described as ‘autistic-like’ (Matson et al. 2013; Luteijn et al. 2000; Mulligan et al. 2009; Santosh and Mijovic 2004; Buitelaar et al. 1999; Greene et al. 1996). Another explanation for social difficulties is the co-occurrence of callous unemotional traits in childhood ADHD. Behavior of children with ADHD may overlap considerably with oppositional defiant disorder or conduct disorder, which in turn aggravates social and emotional impairment (Fowler et al. 2009; Kahn et al. 2012; Nijmeijer et al. 2008).

Social problems of children with ADHD might be related to deficits in social cognition that are often reported in children with ADHD (Uekermann et al. 2010), which may directly or indirectly stem from executive functions deficits (Bora and Pantelis 2016; Hughes 1998; Geurts et al. 2010). Social cognition deficits encompass problems in perceiving emotions in others, for instance affective prosody, facial expression and body posture, which can be regarded as affective social cognition deficits (Uekermann et al. 2010; Maoz et al. 2014). Complex social cognition deficits have been reported in children with ADHD as well, such as problems in the ability to reason about the mental state of others (theory of mind, ToM). A recently published meta-analysis on social cognition in lifespan ADHD reported that adults with ADHD show diminished and less pronounced problems with social cognition than children with ADHD (Bora and Pantelis 2016). Especially ToM (an aspect of complex social cognition) appeared to improve significantly with age, with no evident ToM deficits in adulthood compared to childhood. Deficits in facial emotion recognition (an aspect of affective social cognition) on the other hand did not diminish over time (Bora and Pantelis 2016).

Although ADHD research generally focuses on the childhood period, it has become clear that ADHD can be a lifelong condition, with clinical as well as psychosocial impairments continuing into adulthood (Asherson et al. 2007; Kooij et al. 2010; Canu and Carlson 2007). Studies on adults with ADHD reported friendship problems and poorer social interactions (Young et al. 2003; Kooij et al. 2010), loneliness (Philipsen et al. 2009) as well as poorer intimate relationships and marital adjustment (Eakin et al. 2004). Furthermore, social deficits in adulthood were associated with mood instability and a lack of job and relationship continuity (Kooij et al. 2010). Adults with ADHD also reported experiencing social isolation and a lifetime burden of the disorder (e.g., due to lower educational achievement), and these accumulative problems in social, emotional and occupational areas were linked to a lower reported quality of life (Brod et al. 2012). Very few studies, however, explored performance on objective neuropsychological tests of social cognition in adults with ADHD. Some studies reported problems of adults with ADHD in facial emotion/affect processing and recognition (Ibanez et al. 2011; Marsh and Williams 2006; Miller et al. 2011), whereas others did not find deficits in measures for empathy and social cognition (Bora and Pantelis 2016; Gonzalez-Gadea et al. 2013). If adults with ADHD present with social cognitive deficits, this can have substantial consequences for their quality of social functioning and quality of life. The present study therefore focuses on the relation between social cognitive style, quality of friendships and ADHD symptoms in adults.

While the use of objective neuropsychological measures is valuable for estimating different aspects of social cognitive functions, they are time-consuming and many relevant aspects of our social life are not accessible via objective testing. This is particularly due to the low ecological validity of neuropsychological measures. Therefore, self-report questionnaires represent a valuable approach for the assessment of social cognitive functioning in everyday life. For instance, the quality of friendships can be measured by means of the Friendship Questionnaire (FQ), measuring the social engagement and the enjoyment and importance of friendships (Baron-Cohen and Wheelwright 2004). From the literature on ASD, particularly the Empathy Questionnaire (EQ) (Baron-Cohen and Wheelwright 2004) and the Systemizing Questionnaire (SQ) (Baron-Cohen and Wheelwright 2003) have been described as reliable and valid tools for assessing a person’s social cognitive style (see for a review: Groen et al. 2015). Based on the empathizing–systemizing theory (Baron-Cohen 2009), the EQ and SQ assess two complementary cognitive styles that allow people to efficiently process and react to complex social or non-social information. A person’s cognitive style can on the one hand be relatively empathizing and on the other hand relatively more systemizing, which is characterized by a drive to construct systems, to analyze its variables and to derive its underlying rules (Baron-Cohen 2002). Based on numerous studies, it can be stated that on average, females adopt a more empathizing cognitive style, whereas males adopt a more systemizing cognitive style (see for a review: Groen et al. 2015). A short version of the EQ has also become available, which additionally allows examination of different aspects of empathy by means of the subscale Emotional Empathy (EE) and subjectively experienced complex social cognitive functioning by means of the subscales Cognitive Empathy (CE) and Social Skills (SS) (Groen et al. 2015; Lawrence et al. 2004). Large sex differences have been demonstrated for EE, but only small sex differences for the CE and SS subscales (Groen et al. 2015). The EQ and SQ have been well validated in patients with ASD, who on average present with a strong systemizing and a weak or deficient empathizing cognitive style (Baron-Cohen and Wheelwright 2003; Baron-Cohen and Wheelwright 2004; Berthoz et al. 2008; Wakabayashi et al. 2007; Wheelwright et al. 2006). The extreme male brain (EMB) theory of autism consequently states that ASD can be considered as an extreme male cognitive style (Baron-Cohen 2002, 2009). Since ASD and ADHD show overlap with regard to social dysfunctions, the relation between symptoms of ADHD and the empathizing–systemizing cognitive style is addressed in the present study.

### The present study

The present study was undertaken in the light of the sparse literature on social cognitive functioning in adult ADHD and the easy implementation and high informative value of the EQ-SQ. The aim was to explore the relationship between subjectively reported social cognitive style, quality of friendships and symptoms of ADHD in a typically developing population. Therefore, it was examined whether adults with no formal diagnosis of ADHD but who scored above thresholds in screening instruments indicating clinical levels of ADHD symptomatology (i.e., ‘subclincial ADHD’) differed from matched controls in their reports of social cognitive style. It was hypothesized that people with subclinical ADHD would present with a more ‘autistic-like’ cognitive profile, with lower scores on EQ and FQ and higher scores on SQ and brain type (i.e., a male profile). The subscale scores (EE, CE and SS) were explored in order to get more refined insight into the different aspects of social cognitive style. In order to draw conclusions on the impact of the cognitive profile on the quality of friendships, it was furthermore explored how reports of social cognitive functioning (in interaction with ADHD symptoms) in a community sample relate to self-reports of the quality of friendships.

## Methods

### Participants

#### Community sample

A community sample of 685 adults (270 males and 415 females) in the age of 16–84 years and a mean age of 33 years participated in the survey for this study. This sample was previously included in a psychometric analysis of the Dutch EQ and SQ-R that is described elsewhere (Groen et al. 2015). Thirty-five people indicated having been diagnosed with a mental disorder and 19 indicated using psychopharmaca as a treatment; no information was collected regarding the type of disorder or medication. The participants were recruited via the social networks of the researchers and psychology students that collaborated on the project. They were contacted face-to-face, by e-mail or social media with a request to participate. A computer link to the survey was provided. In the survey, the participants read the informed consent of the study and agreed with participation when they continued with the questionnaire. The project had been approved by the Ethical Committee of Psychology of the University of Groningen, the Netherlands.

#### Participants with subclinical ADHD and matched controls

For determination of people with subclinical ADHD, a selection of participants was made meeting the criteria for a subclinical DSM-5 diagnosis of ADHD by means of the ADHD Rating Scale (see Materials). The criteria of a subclinical DSM-5 diagnosis were fulfilled if the participant scored in the clinical range on the retrospective as well as current items. Conforming to the scoring system by Kooij et al. (2005), a participant scored in the clinical range of an ADHD symptom if a score of 2 = ‘often’ or 3 = ‘very often’ was obtained on an item. A participant fulfilled a subclinical DSM-5 diagnosis when clinical ADHD symptoms were present on ≥5 items measuring inattention and/or ≥5 items measuring hyperactivity/impulsivity on the current version of the ARS. In addition, the participant also needed clinical ADHD symptoms on ≥6 items measuring inattention and/or ≥6 items measuring hyperactivity/impulsivity on the retrospective version of the ARS. In total, 8.2% (*n* = 56) of the sample fulfilled the criteria of a subclinical DSM-5 diagnosis of ADHD based on both the current and retrospective ARS. This prevalence differed significantly between the sexes (*χ* ^2^(1, *n* = 685) = 8.0, *p* = .005), with males showing a higher prevalence (11.9%) than females (5.8%). Categorization in ADHD subtypes revealed that 2.5% of the participants could be typified as the primarily inattentive type (≥5 symptoms of the inattention items and <5 symptoms of the hyperactivity/impulsivity items), 2.8% as the primarily hyperactive/impulsive type (<5 symptoms of the inattention items and ≥5 symptoms of the hyperactivity/impulsivity items) and 2.9% as the combined type (≥5 symptoms of the inattention items as well as ≥5 symptoms of the hyperactivity/impulsivity items). These prevalences did only differ significantly between sexes for the inattentive subtype for which females had a lower prevalence (1.4%) than males (4.1%) (inattention: *χ* ^2^(1, *n* = 685) = 4.7, *p* = .031; hyperactivity/impulsivity: *χ* ^2^(1, *n* = 685) = 0.5, *p* = .472; combined: *χ* ^2^(1, *n* = 685) = 3.7, *p* = .056).

A control group was created by matching the 56 participants with subclinical ADHD to 56 participants of the remainder of the sample that had the same sex, the same student status (part-time, full-time student or non-student) and similar age. The subclinical ADHD group reported significantly more ADHD symptoms than the control group in adulthood (subclinical ADHD: M = 36.0, SD = 9.3 versus control group: M = 16.2, SD = 7.1; F(1111) = 161.0, *p* < .001, *η* ^2^ = .59) as well as childhood (subclinical ADHD: M = 41.9, SD = 10.0 versus control group: M = 16.8, SD = 10.3; F(1111) = 169.6, *p* < .001, *η* ^2^ = .61) with large effect sizes. This indicates that we successfully selected two extreme groups regarding self-reported ADHD symptoms. The matched groups did not differ in age (F(1113) = 0.00, *p* = .984), with a mean of 26.7 years ranging from 17 to 51 years. Both groups contained 32 males and 24 females. The groups did not differ either in their educational level (*χ* ^2^(4, *n* = 114) = 3.7, *p* = .451), their job status (*χ* ^2^(2, *n* = 113) = 0.3, *p* = .870) or their relationship status (*χ* ^2^(4, *n* = 111) = 9.0, *p* = .061).

## Materials

### ADHD rating scale

The Dutch version of the ADHD DSM-IV rating scale (ARS) was used with items on current ADHD symptoms during the last 6 months and retrospective items on childhood ADHD symptoms below the age of 12 years (Kooij et al. 2005). Both the current and retrospective version consisted of 23 items representing the 18 DSM-IV symptoms, of which 5 doubly stated symptoms (i.e., DSM symptoms 1a, 1d, 2a, 2c and 2d) were split into two items. The participant indicated the number that best described their behavior on a 4-point Likert scale (0 = ‘rarely or never,’ 1 = ‘sometimes,’ 2 = ‘often’ and 3 = ‘very often’). The internal consistency of the ARS appeared good, and internal and external validity was previously demonstrated (Kooij et al. 2005, 2008). The ARS allows a computation of a current and retrospective total score, which is the sum of all 23 items. Sub-scores can also be computed for the inattention items and hyperactive/impulsive items separately, which is, respectively, the sum of all inattention items and the sum of all hyperactive/impulsive items. The ARS can furthermore be used to identify people at risk of having ADHD according to the DSM criteria, as described in the Participants section.

### EQ

A Dutch translation of the 40-item EQ plus 20 filler items (Baron-Cohen and Wheelwright 2004) was used (Groen et al. 2015). The EQ consists of statements about empathic skills which are rated by the participant on a 4-point Likert scale (strongly agree, slightly agree, slightly disagree and strongly disagree). The EQ was scored according to the 28-item scoring system with the subscales Emotional Empathy (EE), Cognitive Empathy (CE), Social Skills (SS) and a total EQ scale (Groen et al. 2015). Examples of items for each subscale are: ‘I can easily tell if someone else wants to enter a conversation’ (CE); ‘I really enjoy caring for other people’ (EE); ‘I often find it difficult to judge if something is rude or polite’ (SS). Eleven items were reverse-keyed. The 20 filler items are unrelated to empathy and were not counted in the scoring, e.g., ‘I dream most nights’. The overall internal consistency, test–retest reliability and validity of the 28-item EQ are good (Groen et al. 2015).

### SQ-R

A Dutch translation of the revised version of the SQ (SQ-R) (Wheelwright et al. 2006) was used (Groen et al. 2015). The SQ consists of 75 statements about systemizing skills that are rated on a 4-point Likert scale (strongly agree, slightly agree, slightly disagree and strongly disagree). Examples of items are: ‘I do not tend to remember people’s birthdays (in terms of which day and month this falls)’; ‘When I hear the weather forecast, I am not very interested in the meteorological patterns’; ‘When I have a lot of shopping to do, I like to plan which shops I am going to visit and in what order’. Thirty-six items were reverse-keyed. One total score of the 75 items was used as a measure for systemizing. The overall internal consistency, test–retest reliability and validity of the SQ are good (Groen et al. 2015).

### Brain type

Based on the EQ and SQ-R, a person’s brain type can be computed, which is the standardized difference score of the SQ-R and EQ: D (see for more details on this calculation: Wheelwright et al. 2006). D was calculated as follows: First, the 28-item EQ and 75-item SQ-R scores were standardized for the total group 1 (*n* = 685) using the following formulas E = [EQ–M(EQ)/56] and S = [SQ-R–M(SQ-R)/150], i.e., the individual difference with the group mean was divided by the maximum score on the respective questionnaire. Brain types were calculated by computing the difference between E and S and by normalizing with the factor ½ as is appropriate for an axis rotation: D = [(S–E)/2]. D represents the continuous variable for brain type, in which a positive score indicates brain type S or extreme type S, a negative score indicates brain type E or extreme type E and a score close to zero indicates a balanced brain type (B).

### FQ

A Dutch translation of the Friendship Questionnaire (FQ) was used, which is a 35-item self-report questionnaire measuring the quality of friendships (Baron-Cohen and Wheelwright 2003). A high score on the FQ is achieved by persons experiencing enjoyment and importance of friendships and interest in other people. On each item, the participants have to decide which statement about friendships and social interactions is most applicable to them. Items consist of two, three, four or five statements. Twenty-seven out of 35 items are included in the scoring with a maximum of 5 per item, resulting in a maximum total score of 135. Approximately half of the items are reversed items. The FQ was demonstrated to have high internal consistency (Cronbach’s *α* = .84) and good validity; females achieve higher FQ scores than males, and patients with high-functioning autism achieve lowered FQ scores compared to both male and female controls (Baron-Cohen and Wheelwright 2003).

## Statistical analyses

### Subclinical ADHD

To estimate differences in social cognitive style and quality of friendships between participants with subclinical ADHD and the control group, univariate ANOVAs were performed on the total EQ score and its subscales (CE, EE and SS), the total SQ-R score and D with *group* (subclinical ADHD, controls) and *sex* (male, female) as independent variables. Potential differences between *ADHD subtypes* were explored by performing subgroup analyses with subclinical DSM-5 diagnoses of the combined, inattentive and hyperactive/impulsive type. Effect sizes (Cohen’s d or partial eta-squared) were calculated for all group comparisons to indicate the magnitude of the group differences.

### Predicting FQ

A multiple linear regression was performed on the whole sample of *n* = 685, in order to explore to what extent current symptoms of ADHD, social cognitive style and the combination of these two predict a person’s quality of friendships. FQ score was entered as the dependent variable and the following variables were entered as predictors using the forward method: the ARS total score, the subscale scores of the EQ (CE, EE and SS), the total SQ-R score, D and the interaction terms of the ARS total score and the social cognitive style scores (11 in total). As sex differences in FQ scores have previously been documented (Baron-Cohen and Wheelwright 2003), sex was entered as a covariate by entering it as a fixed predictor in a separate block before entering the other predictors. The interaction terms were computed as the product of the social cognitive style score (CE, EE, SS, SQ and D) and the inverse ARS score (the maximum possible ARS score, i.e., 69, minus the obtained ARS total score). The inverse ARS score was used in order to create a meaningful interaction variable, with high scores reflecting high social cognitive functioning in combination with low ADHD traits and low scores reflecting low social cognitive functioning in combination with high ADHD traits. So as to explore the relation with ADHD subtype, the regression was repeated for the ARS inattention score and the ARS hyperactive/impulsive score.

## Results

### Subclinical ADHD

As can be seen in Table 1, the group with subclinical ADHD had significantly lower scores than the control group on the EQ total scale and the EE subscale, but did not differ significantly on the CE and SS subscales, the SQ-R total score and the FQ total score. Furthermore, the participants with subclinical ADHD on average had a significantly more systemizing brain type, as reflected by a significant difference between groups in D. When inspecting Cohen’s d, the significant group effects had small effect sizes. The absolute scores on the EQ and SQ-R of the subclinical ADHD group as well as the control group fell in the normal range when compared to normative scores of the total sample of *n* = 685 (Groen et al. 2015), with a mean group value lying well above the 10th percentile.

**Table 1:** Means and standard deviations of the social cognitive style measures and quality of friendships for the participants with a subclinical DSM-5 diagnosis of ADHD (subclinical ADHD) and the matched control participants (controls)

	Subclinical ADHD (*n* = 56) M(SD)	Controls (*n* = 56) M(SD)	Main effect *group* F(1108)=	Main effect *sex* F(1108)=	Interaction effect group*sex F(1108)=	Cohen’s d *group* effect	Cohen’s d *sex* effect
EQ total	26.7 (9.6)	30.9 (10.5)	6.3, *p* = .013*	16.7, *p* < .001***	1.9, *p* = .175	−0.42	0.77
EQ CE	11.0 (5.6)	12.3 (5.0)	2.4, *p* = .128	0.7, *p* = .407	2.5, *p* = .125	−0.26	0.15
EQ EE	11.1 (4.7)	13.2 (5.2)	7.7, p = .006**	56.8, *p* < .001***	0.8, *p* = .380	−0.42	1.40
EQ SS	5.9 (2.8)	6.9 (2.5)	3.3, *p* = .073	1.8, *p* = .188	0.1, *p* = .770	−0.38	0.26
SQ-R	59.5 (20.0)	53.7 (17.4)	3.5, *p* = .066	16.4, *p* < .001***	0.6, *p* = .455	0.31	0.80
D	0.074 (0.121)	0.017 (0.121)	8.8, *p* = .004**	28.8, *p* < .001***	2.2, *p* = .145	0.47	1.01
FQ	79.0 (16.0)	78.9 (14.1)	0.0, *p* = .982	16.3, *p* < .001***	0.2, *p* = .656	0.01	0.78

The effect size and test results of the group differences are reported

*EQ* Empathy quotient, *CE* cognitive empathy, *EE* emotional empathy, *SS* social skills, *D* difference between standardized EQ and SQ (‘brain type’), *FQ* friendship questionnaire

* *p* < 0.05; ** *p* < 0.01; *** *p* < 0.001

The typical sex differences were found for all social cognitive measures and the FQ total score, with the exception of the CE and SS subscales of the EQ, which typically show small effects (Groen et al. 2015). No significant interaction effects of *group* and *sex* were found, indicating that the reported main effects of *group* are not influenced by the sex of the participant.

As can be seen in Table 2, the three subtypes of the *ADHD group* did not differ in the social cognitive style measures and the quality of friendships when covaried for sex. These non-significant differences had small to medium effect size.

**Table 2:** Means and standard deviations of the social cognitive style measures and quality of friendships for the participants with different subtypes of the subclinical DSM-5 diagnosis of ADHD (shortly referred to as ‘ADHD subtype’): combined, inattentive and hyperactive/impulsive type

	ADHD combined type (*n* = 20, 12 males) M(SD)	ADHD inattentive type (*n* = 17, 11 males) M(SD)	ADHD hyperactive/impulsive type (*n* = 19, 9 males) M(SD)	Main effect *ADHD subtype* F(2,55)=	Partial eta-squared *ADHD subtype* effect
EQ total	26.8 (12.9)	26.6 (8.4)	26.8 (6.8)	<0.1, *p* = .974	<0.01
EQ CE	10.2 (6.9)	10.9 (4.7)	11.8 (4.8)	0.5, *p* = .627	0.02
EQ EE	12.0 (5.3)	11.5 (4.2)	9.9 (4.6)	3.0, *p* = .06	0.10
EQ SS	5.7 (3.4)	6.8 (2.9)	6.4 (2.2)	0.2, *p* = .785	<0.01
SQ-R	62.7 (20.4)	54.8 (24.6)	60.5 (14.7)	1.0, *p* = .370	0.04
D	0.084 (0.151)	0.060 (0.126)	0.077 (0.081)	0.4, *p* = .680	0.02
FQ	83.1 (17.5)	76.4 (17.4)	77.1 (12.7)	1.3, *p* = .282	0.05

The main effect was covaried for sex because the subgroups differed in their male–female ratio

*EQ* Empathy quotient, *CE* cognitive empathy, *EE* emotional empathy, *SS* social skills, *D* difference between standardized EQ and SQ (‘brain type’), *FQ* friendship questionnaire

### Predicting quality of friendships

Table 3 summarizes the model for the prediction of the FQ total score by the ARS total score, the self-reported social cognitive style measures and their interaction terms for the whole community sample. The reported quality of friendships was best predicted by sex, accounting for 15% of the explained variance, in combination with the EE subscale score of the EQ, which explained an additional 13% of variance. This means that a higher quality of friendships is related to sex (with females reporting higher quality than males) and higher scores on EE (independent of sex). Also SQ-R appeared to significantly contribute to the model, but explained less than 1% of variance in the model.

**Table 3:** Social cognitive predictors of quality of friendships (FQ), with sex as a covariate

Variables	Total FQ score			
	Model 1 *B*	Model 2 *B*	Model 3 *B*	Model 4 *B*
Constant	74.4***	60.4***	65.7***	6.2***
Sex	11.9***	6.3***	5.3***	5.5***
EQ EE		1.2***	1.2***	1.1***
SQ-R			−0.1**	−0.1**
EQ SS*ARS inv total				0.01*
*R* ^*2*^	0.15	0.28	0.29	0.30
*F*	124.2***	133.1***	92.5***	71.2***
Δ*R* ^*2*^		.13	.01	.01
Δ*F*		120.3	8.4	5.7

inv = inverse score; as a first step *sex* was forced into the model as a covariate; as a second step the 11 predictors were entered using the forward inclusion model, of which three significantly predicted the FQ score

* *p* < .05; ** *p* < .01; *** *p* < .001

The total FQ score was significantly predicted neither by the ARS total score nor by interaction terms of the social cognitive style measures with ARS total score. One exception is the interaction term of the (inverse) ARS total score with the SS subscale, which significantly predicted the FQ score, but explained less than 1% of variance in the model.

Thus, on top of sex and EE, people with more systemizing scores and people with more ADHD symptoms combined with a low SS score report lesser quality of their friendships, but these account only for a very small amount of the variance in the model.

## Discussion

This exploratory study on the relation between subclinical ADHD symptoms and self-reported social cognitive functioning is one of the first studies examining self-reported social cognitive style in adult ADHD. We demonstrated that adults with a subclinical DSM-5 ADHD diagnosis reported reduced emotional empathy and a more systemizing cognitive style compared to the control group and that this pattern appeared to be independent of sex and ADHD subtype. The reduced emotional empathy score of the subclinical ADHD group was, however, still within the normal range compared to normative data (above the 10th percentile), which might be explained by the fact that not all of the participants of the subclinical ADHD group would meet the criteria of a formal diagnosis of ADHD. Therefore, this study ought to be replicated in a clinical sample of adult patients with a formal ADHD diagnosis. Moreover, additional measures assessing various domains of social cognition should be included. We do, however, speculate that reduced emotional empathy in people with subclinical ADHD is in accordance with studies showing problems with affective social cognition in patients with ADHD (Kooij et al. 2010; Canu and Carlson 2007). This refers to the basic perception of the emotions of others by adequately interpreting for instance prosody, body language and facial expression. The present study adds to the existing scarce knowledge by showing that even a non-clinical adult population with subclinical ADHD present with lower emotional empathy based on self-report questionnaires.

Analysis of the social cognitive predictors of the quality of friendships (as measured by the FQ) in our community sample revealed that the higher quality of friendships is best predicted by the female gender and by a stronger experience of emotional empathy. Female respondents with higher emotional empathy scores reported higher levels of social engagement, enjoyment and importance of friendships. The self-reported quality of friendships was not related to either ADHD symptoms or the combination of more ADHD symptoms and reduced self-reported social cognitive functioning. Even though our findings did not support a lower quality of friendships in subclinical ADHD, people with ADHD might be at higher risk of having a lower quality of friendships since the literature states that adults with clinical ADHD suffer from friendship problems, poorer social interactions and intimate relationships and loneliness (Young et al. 2003; Kooij et al. 2010; Philipsen et al. 2009; Eakin et al. 2004). Since emotional empathy is correlated with the quality of friendships, we speculate that problems in emotional empathy may (indirectly) affect the quality of friendships in groups with clinical levels of ADHD, despite not finding a direct link between the quality of friendships and subclinical ADHD symptoms in the present study. We suggest that future studies should further investigate this potential relation. When our finding that emotional empathy is an important predictor of self-reported quality of friendships in a community sample would hold true for patients with an ADHD diagnosis, it could be of high value for our knowledge of and treatment of adult ADHD, e.g., to assess and target emotional empathy for improving social outcomes of these patients.

The second finding at first glance appeared to confirm our expectation that people with subclinical ADHD report to have a more systemizing cognitive style compared to the control group. However, people with subclinical ADHD obtained similar scores on the SQ as matched controls. Therefore, the more systemizing cognitive style in this sample is primarily due to the reduced (emotional) empathy and does not represent a typical ASD-like profile, which typically shows overall reduced empathy scores and increased systemizing scores (Baron-Cohen and Wheelwright 2003; Baron-Cohen and Wheelwright 2004; Berthoz et al. 2008; Wakabayashi et al. 2007; Wheelwright et al. 2006). The present study points to a quite specific relation between ADHD symptoms and reduced emotional empathy. This profile therefore appears not to resemble ASD profiles, but rather shows overlap with psychopathic traits. Oliver et al. (2016), for example, described that emotional empathy is not related to autistic traits but is associated with ‘cold-hearted,’ psychopathic traits. Unfortunately, we did not control for comorbidities and thus psychopathic traits in the present study, but these speculations point to the need of examining psychopathological comorbidities as well when examining social cognitive styles in patients with ADHD.

The social cognitive profile that we observed in our study in adults with subclinical ADHD (i.e., diminished emotional empathy but no differences in cognitive empathy) appears to be in line with previous studies on social cognitive functioning in adults with ADHD. Bora and Pantelis (2016) stressed that adults with ADHD persistently show difficulties in affect recognition, but that ToM functioning normalizes into adulthood. Our finding that specifically emotional empathy was reduced in our adult sample is in line with these outcomes. It has been proposed that social cognition deficits are related to other neurocognitive deficits such as inattention, impulsivity and problems with executive functioning, rather than a separate developmental abnormality (Bora and Pantelis 2016). It could be the case that a broader range of neurocognitive functions improve with age and that higher-order functional neural organization is delayed, but that it is (partly) caught up in adulthood (Sripada et al. 2014). Furthermore, an improvement in social cognitive abilities into adulthood is in line with the ‘prefrontal recovery hypothesis’ as described by Halperin and Schulz (2006), proposing that the remission of several higher-order cognitive functions in adult ADHD is related to the neural development of the prefrontal cortex.

### Strengths and limitations

This study presented with several limitations. First, social cognitive style was merely measured by self-report questionnaires which rely on a realistic self-perception. It is possible that people with a subclinical diagnosis of ADHD over- or underestimate their empathic and social skills. For example, several studies have shown that children, adolescents and adults with ADHD show a ‘positive illusory bias,’ as defined by ‘a self-enhancing discrepancy between self-reports of competence and external indices of competence, such as ratings by other informants (e.g. teachers, parents, peers) or objective measures of performance’ (Emeh et al. 2015; Hoza et al. 2010; Prevatt et al. 2012; Volz-Sidiropoulou et al. 2016). However, more realistic and lower levels of self-reflections have also been found in adults with ADHD (Fuermaier et al. 2015). A great limitation of the use of self-report questionnaires in general is that the outcomes might be influenced by these types of biases. Future studies should therefore control for such a bias and/or confirm these findings by the use of objective neuropsychological tests and/or other-report questionnaires. A second limitation is that our study sample included participants with a subclinical diagnosis of ADHD and that the results therefore cannot be directly generalized to clinical samples of patients with ADHD. It is possible that the social cognitive profile of people with clinical ADHD diagnoses differs from the one found in this study. Based on previous studies on social cognition in ADHD, it is expected that in adults diagnosed with ADHD, the effects might be stronger and/or not limited to emotional empathy (Ibanez et al. 2011; Marsh and Williams 2006; Miller et al. 2011). A final limitation is that, based on the measures included in the present study, it remains unclear to what extent the reduced social empathy in the subclinical ADHD group might have been caused by other factors that have been related to reduced empathy, such as traits of autism or callousness.

A major strength of this study is that it is one of the very few studies examining social cognitive functioning in adults with (subclinical) ADHD. As social cognitive functions of adults with ADHD remains a relatively underexposed research field, our study contributes to the scarce existing knowledge on this topic. Furthermore, all participants were assessed with well-validated self-report questionnaires. The fact that we found lower emotional empathy scores in a subclinical ADHD group points to the relevance of replication and extension of this study in clinical ADHD groups.

### Overall conclusion and future directions

Based on self-reported questionnaires in a subclinical, adult ADHD group, we found lower self-perceived emotional empathy scores, independent of sex and ADHD subtype. Self-perceived cognitive empathy and social skills were not diminished compared to the control group, which is in line with a recently published meta-analysis on social cognition in adult ADHD (Bora and Pantelis 2016). Since the emotional empathy score of the subclinical ADHD group was within the normal range and our sample consisted mainly of adults without a formal ADHD diagnosis, this study should be replicated and extended in adults with a clinical ADHD diagnosis. We furthermore suggest that future studies should apply neuropsychological tests rather than only self-report questionnaires as well as tests assessing not only various social cognitive domains but also other neuropsychological domains (e.g., attention, impulsivity, executive functions). The potential relation between emotional empathy and friendship quality in adults with ADHD should be assessed as well. Moreover, comorbidities to ADHD, such as ASD, should be taken into account, in order to find out whether reduced emotional empathy is specific for ADHD or whether it relates more strongly to other traits. Lastly, future studies should explore the relation between emotional empathy and emotion regulation in adults with ADHD. We cautiously suggest that reduced emotional empathy might be related to a broader phenomenon reported in ADHD, namely a reduced emotion regulation/emotional lability in patients with ADHD (Kooij et al. 2010). Emotional lability is reflected by irritable moods with changeable and volatile emotions and has been suggested to be a core dimension of ADHD (American Psychiatric Association 2000; Asherson 2005; Barkley and Fischer 2010; Reimherr et al. 2010; Skirrow et al. 2009; Skirrow and Asherson 2013; Skirrow et al. 2014). Emotional lability is thought to be related to problems in many areas of life (e.g., community activities, school/work, home, Barkley and Fischer 2010; Barkley and Murphy 2010; Skirrow and Asherson 2013) and may be related to reduced (emotional) empathy as well. Future studies should therefore extend our findings by thoroughly assessing a broader range of social cognitive and neurocognitive functions in adults with clinical ADHD.
